# Insights to correlations and discrepancies between impaired lung function and heart failure in Eisenmenger patients

**DOI:** 10.1177/2045894019899239

**Published:** 2020-02-28

**Authors:** Lina Gumbiene, Lina Kapleriene, Dovile Jancauskaite, Monika Laukyte-Sleniene, Elena Jureviciene, Virginija Rudiene, Egle Paleviciute, Mindaugas Mataciunas, Virginija Sileikiene

**Affiliations:** 1 Clinic of Cardiac and Vascular Diseases, Institute of Clinical Medicine, Faculty of Medicine, Vilnius University, Vilnius, Lithuania; 2 Clinic of Chest Diseases, Immunology and Allergology, Institute of Clinical Medicine, Faculty of Medicine, Vilnius University, Vilnius, Lithuania; 3 Department of Radiology, Nuclear Medicine and Medical Physics, Institute of Biomedical Science, Faculty of Medicine, Vilnius University, Vilnius, Lithuania

**Keywords:** pulmonary arterial hypertension, pulmonary function test, bronchial obstruction, adult congenital heart disease

## Abstract

Impaired lung function and spirometric signs of airway obstruction without common risk factors for chronic obstructive pulmonary disease could be found in patients with Eisenmenger syndrome. This study aimed to analyse the association between lung function parameters and disease severity (including heart failure markers, associated congenital heart defect) as well as the possible reasons for airflow obstruction in Eisenmenger syndrome. The data of 25 patients with Eisenmenger syndrome were retrospectively evaluated. The patients were divided into groups according to airflow obstruction and a type of congenital heart defect. Airflow obstruction was found in nearly third (32%) of our cases and was associated with older age and worse survival. No relation was found between airway obstruction, B-type natriuretic peptide level, complexity of congenital heart defect and bronchial compression. Most of the patients (88%) had gas diffusion abnormalities. A weak negative correlation was noticed between gas diffusion (diffusing capacity of the lung for carbon monoxide) and B-type natriuretic peptide level (r = −0.437, p = 0.033). Increased residual volume was associated with higher mortality (p = 0.047 and p = 0.021, respectively). A link between B-type natriuretic peptide and lung diffusion, but not airway obstruction, was found. Further research and larger multicentre studies are needed to evaluate the importance of pulmonary function parameters and mechanisms of airflow obstruction in Eisenmenger syndrome.

## Introduction

Eisenmenger syndrome (ES) is a multisystemic disorder manifesting as the most severe form of pulmonary arterial hypertension (PAH) in patients with congenital heart defects (CHD) developing after longstanding uncorrected significant cardiovascular shunts.^1^ Currently, in developed countries, ES is rare due to available early diagnostic procedures and advanced cardiac surgery, especially in simple CHD.^2^ Furthermore, PAH-targeted therapies prolong ES survival, and these patients reach elderhood and present with more complicated conditions.^3–5^ Due to reverse shunt from pulmonary to systemic circulation through defects at cardiac or great arterial level, ES presents with cyanosis and secondary erythrocytosis.^6^ This condition could cause multiple organ dysfunction, including chronic heart failure (HF) – diastolic and systolic left (not only right – sub-pulmonary) ventricular dysfunction.^7^ It has been reported that up to 60% of patients with chronic HF have ventilation and diffusion abnormalities with reduction of lung volumes on lung function testing.^8^ The diagnosis of chronic obstructive pulmonary disease (COPD) in patients with HF might be challenging, because both conditions may overlap.^9–11^ There is a controversy about changes in lung function in pulmonary hypertension (PH), especially in patients with ES, because the precise mechanisms that contribute to abnormal changes of lung function are not clearly known, probably are multifactorial and variable, depending on the underlying CHD.^12,13^

The aim of the study was to determine the relationship between lung function and disease severity (including HF markers and associated CHD) and to analyse the possible causes of airflow obstruction in patients with ES.

## Methods

A retrospective analysis of our hospital PH database was performed. Adult patients (age ≥18 years) with the diagnosis of ES, who underwent comprehensive pulmonary function tests (PFT), including spirometry, body plethysmography and gas diffusion evaluation (diffusing capacity of the lung for carbon monoxide – DLCO) were included. All patients had been on stable medical therapy for at least three months at the time of PFT. Patients were divided into two groups according to the presence of airflow obstruction (FEV1/FVC < 70% and < lower limit of normal (LLN)): with airflow obstruction and without it. Reversibility of airway obstruction was considered positive if FEV_1_ and (or) FVC improved >12% and 200 ml after 400 µg salbutamol inhalation. The patients of these two groups were compared by following parameters: CHD type, medications, arterial blood gases, hemoglobin level (HgB), B-type natriuretic peptide (BNP) level, the distance walked during six-minute walk test (6 MWT), signs of airway compression on computed tomography (CT) images and survival. CT images were acquired during maximal inspiration. Prior the CT image acquisition, patients were trained and monitored to perform maximal breath-hold in maximal inspiration correctly. CT images were evaluated visually by a single experienced thoracic radiologist and rated qualitatively as positive or negative for bronchial compression. CHDs were divided into simple (one simple pre-tricuspid or post-tricuspid lesion), combined (several simple defects) and complex groups based on anatomy.^14^ Combined and complex CHD patients were merged in one combined/complex CHD group due to a small number of individuals in these groups. Based on the hospital electronic patient’s records system and national health insurance data, survival status was assessed.

Data were presented as a mean ± standard deviation (SD) or median (interquartile range (IQR)) for continuous variables. Categorical variables were described as absolute numbers and percentages. The Mann-Whitney U and Independent-Samples T tests were used for the comparison between the groups. The Chi-Square or Fisher’s exact tests were used to compare categorical variables. The association between BNP level and lung diffusion was evaluated with linear regression. Statistical analyses were performed using IBM SPSS Statistics version 23.0. A p-value less than 0.05 was considered statistically significant.

## Results

From 405 adult patients with PH included in the database, 75 patients with PAH due to CHD were found, and 36 patients with ES were selected. Twenty-five ES cases with comprehensive PFT and chest CT data were eligible for the final analysis.

The mean age of these patients was 42.0 ± 12.2 years. Most of the patients (64%) were female. More than two-thirds (68%) of the cases were in World Health Organization functional class (WHO-FC) III. All except one (96%) received disease-targeting PAH therapy: 15 (60%) patients were on monotherapy and 9 (36%) on combination therapy. Gas diffusion abnormalities were particularly common; DLCO level was < LLN in 22 (88%) patients.

According to spirometry, airflow obstruction was found in eight patients (32%). These patients were older than those without obstruction (54.5 and 32.0 years, p < 0.001). Reversible airway obstruction was detected in two patients (25% of all obstructive groups). All patients were non-smokers, with the exception of the only smoker, who had bronchial obstruction.

**Fig. 1.**
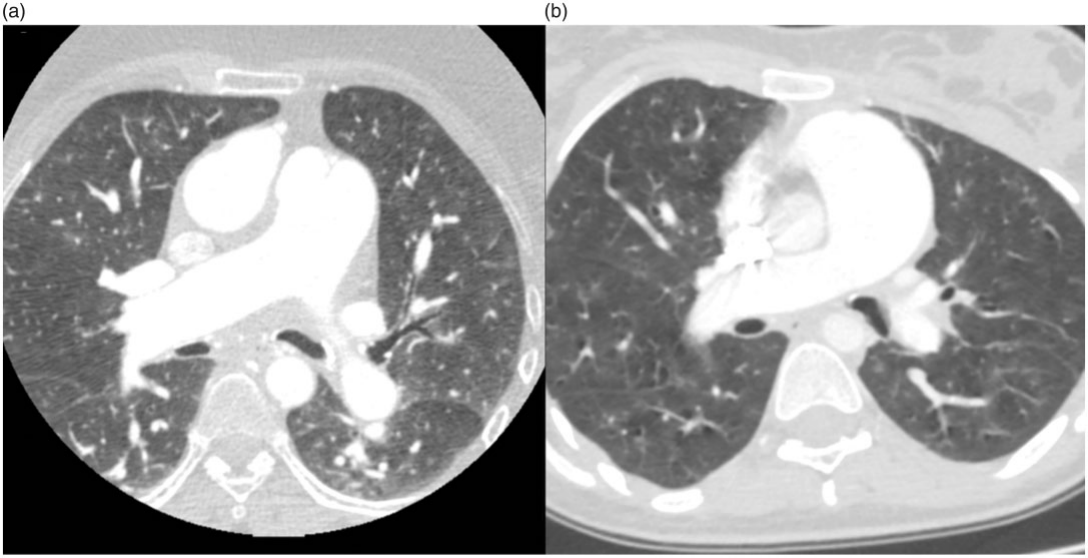
Chest CT axial slices at the level of the main right and left bronchi during maximal inspiration showing no signs of compression by enlarged pulmonary artery branches. (a) Patient with clinically proved airflow obstruction and (b) patient without airflow obstruction.

Sixteen (64.0%) patients were on treatment with beta-adrenoblockers. No relationship was found between airflow obstruction, BNP, DLCO, 6 MWT distance, HgB, arterial blood gasses, the use of beta blockers and bronchial compression on CT (Table 1, Fig. 1). There was no other significant difference comparing obstructive and non-obstructive groups in PFT parameters such as forced vital capacity (FVC), forced expiratory volume in one second (FEV1), total lung capacity (TLC) and DLCO. Four patients died during a follow-up period of 0.7–4.5 (2.4) years. The mortality was observed only in the airflow obstruction group. All four lethal cases had simple CHD, one of them pre-tricuspid CHD, the mean age during death was 56.8 ± 2.8 years. The reasons for their death were: pulmonary embolism, pulmonary bleeding, obturating tumor of the right atrium and sudden death.

**Table 1.** Data comparison between the groups depending on the presence of airflow obstruction.

Variable	Total (n = 25 (100%))	Without airflow obstruction (n = 17 (68%))	With airflow obstruction (n = 8 (32%))	p
Age (years)	45.0 [25.0]	32.0 [18.0]	54.50 [9.0]	**<0.001**
Sex, n (%)				
Male	9 (36.0)	5 (29.41)	4 (50.0)	0.394
Female	16 (64.0)	12 (70.59)	4 (50.0)	
CHD, n (%)				
Simple	14 (56.0)	8 (47.06)	6 (75.0)	
Combined/complex	11 (44.0)	9 (52.94)	2 (25.0)	0.234
WHO-FC, n (%)				
I	0 (0)	0 (0)	0 (0)	0.330
II	7 (28.0)	5 (29.41)	2 (25.0)	
III	17 (68.0)	12 (70.59)	5 (62.50)	
IV	1 (4.0)	0 (0)	1 (12.50)	
Treatment with beta-adrenoblockers, n (%)	16 (64.0)	11 (64.71)	5 (62.50)	0.626
Mortality, n (%)	4 (16.0)	0 (0)	4 (50.0)	**0.006**
HgB (g/L)	183.57 (±30.81)	184.41 (±33.78)	181.79 (±25.29)	0.847
pO_2_ (mmHg)	53.59 (±12.18)	52.53 (±9.40)	55.84 (±17.26)	0.538
pCO_2_ (mmHg)	36.48 (±9.13)	35.39 (±5.05)	38.81 (±14.77)	0.393
BNP (ng/L)	160.71 (±135.11)	135.31 (±127.77)	214.69 (±142.75)	0.176
SO_2_ (%)	83.83 (±7.80)	83.57 (±6.73)	84.38 (±10.21)	0.816
6 MWT distance (m)	450.12 (±66.13)	450.0 (±71.55)	450.38 (±57.38)	0.990
FVC (%)	78.56 (±18.83)	73.06 (±16.02)	90.25 (±19.98)	0.055
FEV1 (%)	67.0 [21.0]	69.0 [14.0]	67.0 [26.0]	0.770
FEV1/FVC (%)^a^	70.84 (±12.22)	77.12 (±7.92)	57.50 (±8.32)	**<0.001**
TLC (%)	89.0 [17]	86.0 [14.0]	93.50 [18.0]	0.075
VC (%)	82.54 (±18.31)	76.75 (±14.75)	94.13 (±20.14)	**0.025**
RV (%)	106.0 [47.0]	97.0 [42.0]	117.0 [57.75]	0.101
DLCO (%)	64.84 (±11.21)	65.18 (±10.38)	64.13 (±13.54)	0.832

Note: The data are presented as number (percentage), or mean (±SD), or median value [IQR].

6 MWT: six-minute walk test; BNP: B-type natriuretic peptide; CHD: congenital heart defect; DLCO: diffusing capacity of the lung for carbon monoxide; FEV1: forced expiratory volume in one second; FEV1/FVC: forced expiratory volume in one second/forced vital capacity; FVC: forced vital capacity; HgB: hemoglobin; pCO_2_: partial pressure of carbon dioxide; pO_2_: partial pressure of oxygen; RV: residual volume; SO_2_: oxygen saturation; TLC: total lung capacity; VC: vital capacity; WHO-FC: World Health Organization functional class.

a In airway obstruction group FEV1/FVC was < lower limit of normal (LLN).

The simple CHD group consisted of 14 ES patients, predominantly with ventricular septal defect (VSD) (9 patients (64.3%) with VSD), 2 (14.3%) patients had atrial septal defect (ASD) and 3 (21.4%) had patent ductus arteriosus (PDA). The combined/complex CHD group included patients of ASD and VSD (n = 3 (27.3%)), VSD and PDA (n = 3 (27.3%)), VSD and transposition of great arteries (n = 2 (18.2%)) and other defects (n = 3 (27.3%)). Patients with combined/complex CHD were younger than patients with simple defects (Table 2). In the combined/complex CHD group, oxygen saturation (measured by pulse oximeter at rest) and BNP levels were worse (p = 0.038 and p = 0.041, respectively) comparing with simple CHD patients (Table 2). No relationship among PFT parameters, 6MWT distance, WHO-FC, HgB levels, arterial blood gases value and the complexity of heart defect was found.

**Table 2.** The data comparison between the groups according to underlying CHD.

Variable	Simple CHD (n = 14 (56%))	Combined/complex CHD (n = 11 (44%))	p
Age (years)	48.50 [12]	31.0 [17]	0.013
Sex, n (%)			
Male	8 (57.1)	1 (9.1)	0.033
Female	6 (42.9)	10 (90.9)	
BNP (ng/L)	112.49 (±85.75)	222.08 (±163.87)	0.041
SaO_2_ (%)	86.66 (±7.28)	79.5 (±7.13)	0.038

Note: The data are presented as number (percentage), or mean (±SD), or median value [IQR].

BNP: B-type natriuretic peptide; CHD: congenital heart defect; SaO_2_: oxygen saturation.

There was a weak but significant negative correlation between DLCO and BNP level (r = −0.437, p = 0.033). Increased residual volume (RV) was associated with higher mortality (101.3 ± 23.6 in alive patients and 146.3 ± 34.4 in deceased patients, p = 0.021).

## Discussion

Our study confirms that lung function impairment is very frequent in adult patients with ES. Gas diffusion abnormalities were found in more than 80% of our cases and obstruction in one-third of the patients. Moreover, right ventricle dysfunction assessed by BNP levels was associated with pulmonary gas diffusion, but not with airflow obstruction or lung restriction parameters at PFT in our cohort. A higher RV correlated with poor survival in ES patients.

A few publications analysing the value of pulmonary function and gas exchange in ES were published^8,10,11,15^ and revealed lung gas diffusion abnormalities in 47–80% of cases. Ventilation and diffusion abnormalities can be caused by several factors, such as respiratory muscle weakness, lung fluid imbalance, pulmonary hypertension and/or chronic interstitial edema, resulting in pulmonary membrane thickening and fibrosis.^8,16^ In a study by Broberg et al.,^10^ airflow obstruction was observed in 41% of patients with ES, between our patients this prevalence was slightly lower – 35%. A tendency towards increasing airflow obstruction with age was observed by both ourselves and Broberg et al.^10^ Despite the fact that some small paediatric studies have shown benefit with inhaled bronchodilators in patients with PH,^17,18^ our study showed that only 25% of our patients with obstruction had a positive response to salbutamol, although FEV_1_/FVC maintained reduced <LLN (obstruction did not completely disappear).

But can airway obstruction in ES patients be called COPD? According to the guidelines, the diagnosis of COPD is based on respiratory symptoms (cough, expectoration, dyspnea), exposure to tobacco smoke or other noxious agents and evidence of airflow obstruction, which was confirmed by spirometry.^18^ Lung function parameters could show obstruction in some ES patients, but most of these patients have neither typical COPD clinical signs, nor common risk factors. Furthermore, we have empirically noticed that standard COPD treatment with bronchodilator therapy is ineffective in our patients. The cause of airway obstruction in ES seems to be multifactorial: loss of elastic recoil at low lung volumes, intrinsic narrowing or obliteration of small airways, effect of vasoactive and inflammatory mediators and mechanical encroachment of dilated vessels.^10,11,15,20–23^ Increased serum endothelin-1 level is correlated with airway obstruction in CHD-associated PAH.^11^ Airflow obstruction in PH may be due to compression of the mainstem bronchi by dilated pulmonary arteries.^21–23^ Unfortunately, we cannot confirm this hypothesis because in our study, bronchial compression on CT did not differ in the obstruction and non-obstruction groups (Fig. 1).

Combining spirometry, DLCO and BNP levels may generally allow differentiation between heart and pulmonary disorders in patients with dyspnea,^25^ but this approach is not appropriate in ES. Gas diffusion impairment in these patients could be due to a reduction in pulmonary capillary blood volume and pulmonary membrane diffusion capacity as a consequence of increased pulmonary vascular resistance, reduced cardiac output and endothelial cell proliferation.^11,26^

Elevation of cardiac biomarker BNP is associated with poorer prognosis and disease progression in patients with CHD, including ES.^28^ Şahingözlü et al. confirmed that plasma BNP levels were affected much more in cardiac pathology rather than in the lung disease,^29^ but there is a lack of studies about BNP association with lung function parameters. Hawkins et al.’s systemic review showed that natriuretic peptide levels are always significantly elevated in patients with COPD and concomitant HF or left ventricle systolic dysfunction compared to those without.^30^ In our cohort, airflow obstruction was not associated with BNP level or complexity of CHD, but half of the patients in the obstruction group died during follow-up.

DLCO correlated negatively with BNP level, as we expected. This finding coincides with the work of other researchers: it has been recently shown that DLCO is significantly lower in patients with HF with preserved ejection fraction as well.^31^ The most likely cause of this phenomenon could be the alveolar-capillary membrane thickening and/or alveoli space filling with transudate.

Another interesting finding is that increased RV of the lung was associated with worse survival. RV is the volume of air that remains in the lungs after maximum forceful expiration. The RV function is to keep the alveoli open even after maximum expiration.^32^ In healthy lungs, the air that makes up the RV allows for continual gas exchange to occur between breaths. The oxygen-depleted residual air is then mixed with newly inhaled air to improve gas exchange at the alveoli. In obstructive lung diseases (OLD), such as COPD, asthma or bronchiectasis, inflammation and decreased elastic recoil increase airway resistance and lead to earlier small airway closure during expiration. That is, the pleural pressure exceeds the airway pressure earlier, trapping air in the lungs. This trapped air results in pulmonary hyperinflation. Patients with OLD often have increased TLC, FRC and RV.^33^ Our results did not show the difference between TLC and RV in patients with airflow obstruction and those without. This fact suggests that our obstructive ES patients did not have all typical signs of lung volumes damage usually present in COPD.

The severity of reduced forced vital capacity in unrepaired/palliated CHD relates to the complexity of underlying CHD, cardiothoracic ratio and presence of scoliosis.^8^ However, a reduced FVC and the prevalence of bronchial obstruction were not associated with the complexity of underlying CHD in our study.

Symptoms of congestive HF are usually mild in patients with ES due to right ventricle compensation and adaptation through the years.^14,33^ Nevertheless, signs and symptoms of HF, that usually develop in later stages of the disease, are associated with increased mortality in ES patients as well as older age.^34,35^ Hypoxia is one of the most important factors determining the increase of BNP in unrepaired CHD.^36,37^ Therefore, more complex CHD could lead to more severe desaturation and increase in BNP among patients with ES, as it was observed in our study.

### Limitations

This is a single-centre retrospective study. Due to different reasons, not all ES patients could undergo complete PFT; therefore, they were not included for the final evaluation. A rather small group of patients with a rare disease could have an impact on statistically significant differences in our calculations. Not all blood samples for BNP, HgB were taken and 6 MWT was performed at the same day as PFT due to the retrospective design of the study. Additional prospective multicenter studies could provide a more accurate assessment of pulmonary function in patients with ES.

## Conclusions

Airflow obstruction and lung gas diffusion abnormalities were common in ES patients. A link between BNP and lung diffusion but not with airway obstruction was found. Increased residual volume was associated with a worse prognosis. The exact causes of airflow obstruction in ES patients remain unclear.
